# Flexible Positive Temperature Coefficient Composites (PVAc/EVA/GP-CNF) with Room Temperature Curie Point

**DOI:** 10.3390/polym16142028

**Published:** 2024-07-16

**Authors:** Chao Du, Yangyang Zhang, Jiangmin Lin, Guotao Fan, Can Zhou, Yan Yu

**Affiliations:** 1 Union Hospital Tongji Medical College and School of Integrated Circuits, Huazhong University of Science and Technology, Wuhan 430074, China; 2 Henan Key Laboratory of Nanocomposites and Applications, Huanghe Science and Technology College, Zhengzhou 450061, China

**Keywords:** polymeric positive temperature coefficient, room temperature Curie Point, EVA and PVAc blends, graphite and CNT co-fillers

## Abstract

Polymeric positive temperature coefficient (PTC) materials with low switching temperature points are crucial for numerous electronic devices, which typically function within the room temperature range (0–40 °C). Ideal polymeric PTC materials for flexible electronic thermal control should possess a room-temperature switching temperature, low room-temperature resistivity, exceptional mechanical flexibility, and adaptive thermal control properties. In this study, a novel PTC material with a room-temperature switching temperature and superb mechanical properties has been designed. A blend of a semi-crystalline polymer EVA with a low melting temperature (Tm) and an amorphous polymer (PVAc) with a low glass transition temperature (Tg) was prepared. Low-cost graphite was chosen as the conductive filler, while CNF was incorporated as a hybrid filler to enhance the material’s heating stability. PVAc0.4/EVA0.6/GP-3wt.% CNF exhibited the lowest room temperature resistivity, and its PTC strength (1.1) was comparable to that without CNF addition, with a Curie temperature of 29.4 °C. Room temperature Joule heating tests revealed that PVAc0.4/EVA0.6/GP-3wt.% CNF achieved an equilibrium temperature of approximately 42 °C at 25 V, with a heating power of 3.04 W and a power density of 3.04 W/cm^2^. The Young’s modulus of PVAc0.4/EVA0.6/GP-3wt.% CNF was 9.24 MPa, and the toughness value was 1.68 MJ/m^3^, indicating that the elasticity and toughness of the composites were enhanced after mixing the fillers, and the mechanical properties of the composites were improved by blending graphite with CNF.

## 1. Introduction

Conductive polymer composites (CPCs) are of widespread interest due to their versatility and dexterity in various engineering applications, such as in overcurrent protectors, electronic devices, electromagnetic shielding, flexible sensors, and self-regulating heaters [1,2,3,4,5,6,7]. Specifically, certain CPCs exhibit a thermal resistance behavior, whereby the resistivity increases sharply when the temperature exceeds a certain point, referred to as the switching temperature. This is attributed to the severe thermal expansion near the melting point of the polymer matrix, resulting in an increase in the spacing between conductive particles. This phenomenon is defined as the positive temperature coefficient (PTC) effect and is caused by the mismatch of thermal expansion between the polymer matrix and the filler, as well as by the disruption of the conductive network [8,9]. In general, above the melting point, the resistivity decreases inversely with increasing temperature, exhibiting a negative temperature coefficient effect (NTC) [10]. However, the NTC effect can significantly impact the performance of PTC materials due to the aggregation of conductive particles and the random reconfiguration of the conductive network.

To date, there have been numerous attempts to obtain PTC materials with superior performance, but most of the research has concentrated on the high temperature region, with switching temperatures ranging from 50 to 400 °C [11,12,13,14,15]. However, in numerous regions, the operating temperature range of electronic devices is often required to be within the room temperature range of 0–40 °C [16,17,18]. To achieve effective temperature control, PTC materials should possess a switching temperature slightly below the control temperature of the electrical device. Therefore, preparing PTC materials with a low switching temperature ranging from 0 to 40 °C has been a crucial issue [19,20,21].

Ideal polymeric PTC materials for thermal control should possess room-temperature switching temperatures [22,23,24], low room-temperature resistivity [25,26,27,28,29], good PTC repeatability [14], high PTC strength [19], good mechanical flexibility [30,31,32,33], and adaptive thermal control properties [21]. It has been demonstrated that PTC materials with low Curie temperatures can be achieved using low melting point semi-crystalline polymers and low glass transition temperature amorphous polymers as matrix materials. These materials exhibit excellent PTC repeatability and mechanical properties, with PVAc0.4/EVA0.6/GP composites showing the best Joule heating performance, yet their heating stability requires improvement. For polymer-based PTC composites to be practical, heating stability is crucial, directly affecting the material’s service life. To enhance heating stability, focus should be on improving PTC repeatability while avoiding exceeding the material’s tolerance temperature for extended periods, as this can also shorten its lifespan. Numerous studies have been conducted to improve PTC repeatability, primarily through filler modification [34,35], hybrid fillers [34,36], polymer blending [37,38,39,40], and macroscopic assembly of different PTC materials [41,42,43]. The current study considered polymer blending to enhance PTC reproducibility during the initial design phase, and the results validated this approach. However, further performance enhancement necessitates considering other methods. Modified fillers can improve compatibility between fillers and polymers, preventing large agglomerates and allowing conductive fillers to be reversibly repositioned. The polymer matrix bonds to filler surface-grafted molecules through physical entanglement or chemical bonding. However, due to the complexity of filler modification and potential conductivity losses with increasing grafting modifiers, this strategy is rarely implemented in practical products. Rational device structure design can also enhance PTC performance, but the manufacturing process is intricate, increasing costs and limiting applications in scenarios requiring lighter, simpler materials, such as aircraft wing de-icing or wearable devices. Hybrid fillers leverage the synergy between fillers to hinder cluster formation. The second filler connects conductive filler clusters over long distances, increasing the likelihood of conductive network formation. This approach often reduces room temperature resistivity while enhancing PTC repeatability. High aspect ratio fillers have been reported to restrict other filler movements, inhibiting the NTC effect [44].

In this paper, carbon nanofibers (CNF) with high aspect ratios were chosen as the second conductive filler to be added to the optimal PVAc0.4/EVA0.6/GP composite in order to further enhance the heating stability of the material. Additionally, the heating stability of the material was tested at low temperatures (<0 °C). The material’s heating performance at low temperatures (<0 °C) was also examined to explore its potential for de-icing applications.

## 2. Experimental

### 2.1. Materials

Graphite (average size about 6.5 μm, density 2.2 g/cm^3^) was purchased from Shenzhen Jinda Power Technology Co. (Shenzhan China) PS (density 1.047 g/cm^3^, melt flow index 7.9–8.9 g/10 min, 200 °C/5 kg) was provided by Shanghai Aladdin Biochemical Technology Co. EVA (40 w, Mr = 70,000–120,000, density 0.965 g/cm^3^, melt flow index 52 g/10 min, 190 °C/2.16 kg) was purchased from DuPont de Nemours & Company (Guangzhou, China). Toluene (AR, Mr = 92.14) was produced by Shanghai Tianteng Technology Co. (Shanghai, China) CNF (purity > 70%, OD 200–600 nm, length 5–50 μm) was provided by Shanghai Aladdin Biochemical Technology Co. (Shanghai, China).

### 2.2. Sample Preparation

The PVAc/EVA/GP-CNF composites were prepared using the solution blending method. Firstly, a 2/3 mass ratio of PVAc and EVA (total mass 0.4 g) was added to 2.9 mL of toluene at the same time, followed by magnetic stirring at 60 °C for 12 h until the polymer was completely dissolved. After being dissolved, 20 wt.% of graphite as well as mass ratios (1 wt.%, 2 wt.%, 3 wt.%, 4 wt.%, and 5 wt.%) of CNF were weighed and added to the mixed solution, and then the multi-step cyclic dispersion method was performed: magnetic stirring for 1.5 h, sonication for 10 min, oscillation on a vortex mixer for 1 min, and then magnetic stirring for 10 min, sonication for 10 min, oscillation on a vortex mixer for 1 min. Immediately after the dispersion process, the mixed solution in the bottle was sucked up with a rubber-tipped dropper and squeezed out onto a clean glass sheet, which was naturally stretched on the surface using the tension of the liquid. The glass sheet was put into an oven at 60 °C and kept warm for 24 h to allow the solvent to evaporate. After the end of the holding period, it was cooled to room temperature and then annealed: the temperature was slowly increased to 70 °C for 10 min, and then cooled to room temperature. Finally, the sample was obtained by stripping the film, and the thickness of the sample was about 200 μm. The composition of the polymer and conductive filler used in the experiment is shown in Table 1.

### 2.3. Characterization

The microscopic morphology of the fracture surface and the distribution of graphite in the co-polymer were characterized by field emission scanning electron microscopy. The fracture surfaces of the samples were obtained by breaking the samples after immersion in liquid nitrogen for 5 min. Gold spraying was performed on all studied surfaces before testing.

The thermal properties of PTC materials were determined by differential scanning calorimetry. A sample weighing about 5 mg was heated from 10 °C to 180 °C at a heating rate of 10 °C/min, held at this temperature for 5 min to remove the thermal history, and then cooled from 180 °C to 10 °C at a cooling rate of 10 °C/min, held for 5 min, and then heated from 10 °C to 180 °C at a rate of 10 °C/min.

The DC resistance of the sample along the thickness direction was measured continuously using the PTCR-T characteristic test system with a heating rate of 2 °C/min. The resistivity ρ was calculated as ρ=RS/d, where *R*, *S*, and d represent the resistance, area, and thickness of the sample, respectively. The sample was cut to a size of 10 mm × 10 mm before testing, and the upper and lower surfaces of the material were sprayed with gold.

A DC power supply was used to apply a voltage to the samples, and the Joule heating properties of the materials at different voltages were tested by varying the voltage and testing their electrical heating stability. The mechanical properties of the samples were tested using an Instron tensile testing machine to evaluate their mechanical properties.

## 3. Results and Discussion

### 3.1. Microscopic Morphological Analysis of PVAc0.4/EVA0.6/GP-CNF Conductive Composites

**Figure 1:**
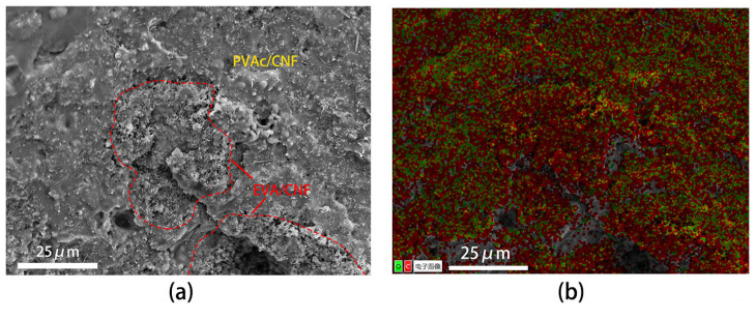
PVAc0.4/EVA0.6/20wt.% CNF: (**a**) SEM image of cross section; (**b**) EDS surface image (the Chinese characters in the figure mean: “Digital Image”).

Figure 1 shows the cross-sectional view of PVAc0.4/EVA0.6/20 wt.% CNF and the corresponding energy dispersive spectroscopy (EDS) image. When the PVAc/EVA mass ratio is 2/3, the morphology of the composite undergoes significant changes when graphite is replaced with the same content of CNF. It has been observed that the region with relatively high O content in the EDS image is primarily PVAc, while the region with low O content is primarily EVA, thus enabling the distinction of regions in Figure 1a based on the EDS (Figure 1b). Initially, when the polymer matrix components were identical, the two phases of the biphasic polymer lost their longitudinal co-continuity after the filler was changed to CNF, resulting in a more distinct division between the EVA and PVAc phases. The EVA phase filled with CNF was more porous and loose, with finer and rougher pores overall. Meanwhile, some pores were observable in the PVAc phase filled with CNF, but they were coarser, and the PVAc phase in the cross-section appeared relatively smoother. Additionally, there were differences in the pattern of CNF filling in the two phases. In the EVA phase, the CNF was smaller, while in the PVAc phase, not only were there some fine CNF interspersed, but also some relatively large CNF tips were visible through the PVAc phase, leading to larger holes in this phase. The reason for this morphological difference may be that EVA is a semi-crystalline polymer, comprising both crystalline and amorphous phases. The introduction of CNF disrupts the crystalline phase structure, resulting in the formation of many loose pores. Conversely, PVAc is a pure amorphous phase with better affinity for CNF, leading to CNF being wrapped in the polymer, resulting in narrower voids and fewer pores.

**Figure 2:**
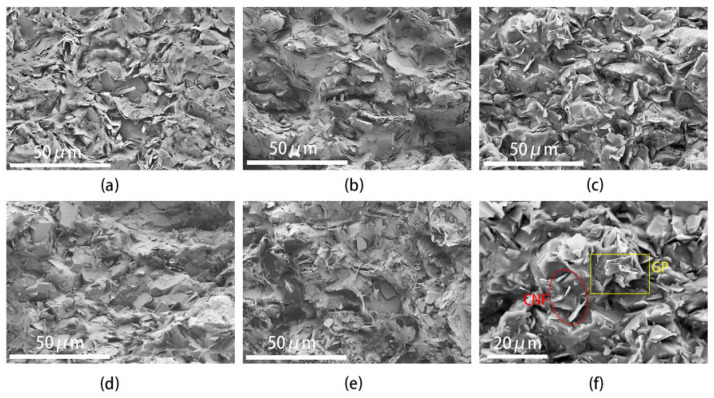
SEM images of PVAc0.4/EVA0.6/GP-CNF composites: SEM images of CNF content of (**a**) 1 wt.%; (**b**) 2 wt.%; (**c**) 3 wt.%; (**d**) 4 wt.%; and (**e**) 5 wt.%. (**f**) Local amplification of (**c**), respectively.

Figure 2 shows SEM cross-sectional images of the PVAc0.4/EVA0.6/GP-CNF composites at different CNF contents. From the figures, it can be seen that the addition of a small amount of second filler CNF on top of 20 wt.% graphite does not significantly affect the morphology of the composites, and the biphasic polymers still exhibit a co-continuous state at these five CNF contents, with no obvious dividing line or partition between the two phases.

**Figure 3:**
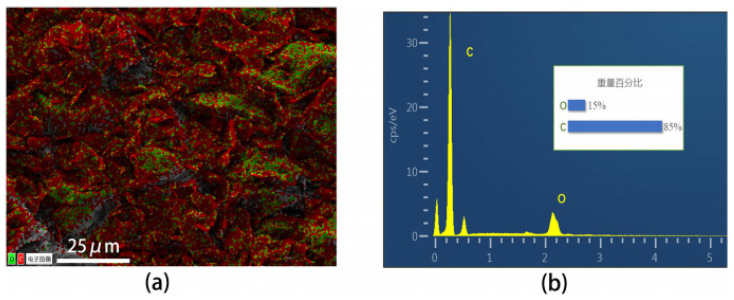
PVAc0.4/EVA0.6/GP-3wt.%CNF composite: (**a**) EDS image: green indicates the distribution of O elements, red indicates the distribution of C elements (the Chinese characters in the figure mean: “Digital Image”).; (**b**) EDS energy spectrum (the Chinese characters in the figure mean: “wt.%”).

According to Figure 3, the distribution of PVAc and EVA phases is similar to that of graphite in the absence of CNF addition. It can be observed that the graphite is well dispersed in the matrix regardless of the CNF content. Graphite plays a significant role in the formation of the conductive network, while CNFs of varying sizes function as a “bridge” connecting graphite particles and forming graphite–CNF–graphite channels. Additionally, some CNFs are directly or indirectly in contact with each other, forming CNF–CNF networks (see Figure S1 in Supporting Information), thus creating a dual conductive network and enhancing the likelihood of forming a conductive pathway. Figure 2f depicts a partial enlargement of the cross-section at a CNF content of 3 wt.%. Notably, CNFs can traverse the polymer phase, serving as a bridge between two-phase regions, which can compensate for the smaller size of graphite particles that may not form long conductive channels alone. This indicates that graphite and CNFs have a synergistic effect on the formation of the conductive network, resulting in a more stable and superior conductive network.

In order to predict the preferential distribution of graphite in the binary-polymer, classical thermodynamics is used. When the equilibrium state is reached, the graphite is either distributed in a specific phase or at the interface of two immiscible polymers, which is dictated by the minimum interfacial energy [45]. The wetting coefficient (ω_a_) can assess the equilibrium state of the conductive fillers based on Young’s equation, shown as Equation (1) [46]:

(1) $$\omega_{a}=\frac{\gamma_{CA}-\gamma_{CB}}{\gamma_{AB}}$$

where γ_CA_, γ_CB_, and γ_AB_ are the interfacial energies between polymer A and the filler, polymer B and the filler, and between polymer A and polymer B, respectively. The values of ω_a_ > 1, ω_a_ < −1, or −1 < ω_a_ < 1 mean that the fillers would preferentially be localized in polymer B, in polymer A, or at the interface, respectively [47]. 

The interfacial energies can be calculated from the surface energies of the dispersion and polar parts. According to the type of surfaces, two main approaches are often used to calculate γij, including the harmonic mean equation and the geometric mean equation [48]. 

Harmonic mean equation:

(2) $$\gamma_{12}=\gamma_{1}+\gamma_{2}-4(\frac{\gamma_{1}^{d}\gamma_{2}^{d}}{\gamma_{1}^{d}+\gamma_{2}^{d}}+\frac{\gamma_{1}^{p}\gamma_{2}^{p}}{\gamma_{1}^{p}+\gamma_{2}^{p}})$$

Geometric mean equation:

(3) $$\gamma_{12}=\gamma_{1}+\gamma_{2}-2(\sqrt{\gamma_{1}^{d}\gamma_{2}^{d}}+\sqrt{\gamma_{1}^{p}\gamma_{2}^{p}})$$

where γ_1_ and γ_2_ are the surface tensions of components 1 and 2; $\gamma_{1}^{d}$ and $\gamma_{2}^{d}$ are the dispersive parts of the surface tensions of components 1 and 2; and $\gamma_{1}^{p}$ and $\gamma_{2}^{p}$ are the polar parts of the surface tension of components 1 and 2. 

The results of the interfacial energy obtained using Equations (2) and (3) are presented in Table 2, and the results of the wetting coefficient are shown in Table 3. The wettability coefficients calculated using the harmonic mean equation and the geometric mean equation are 0.3 and 0.4, respectively, suggesting that graphite is primarily situated at the interface between EVA and PVAc, which aligns with our observed findings. However, for CNF, the wettability coefficients determined by the harmonic mean equation and the geometric mean equation are −2.9 and −3.2, respectively, implying that CNF predominantly resides in the EVA phase. Nevertheless, this inference contradicts the observed packing distribution.

### 3.2. Influence of Mixed Fillers on Electrical and Thermal Resistance Properties

**Figure 4:**
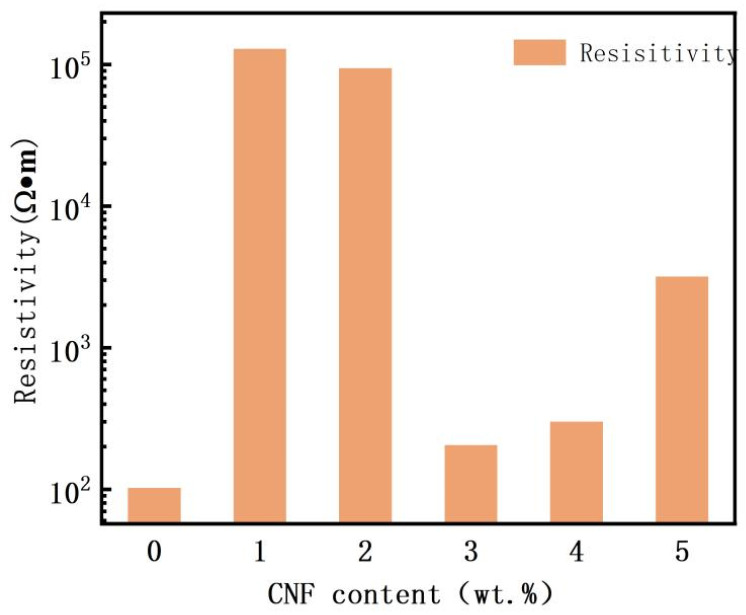
Room temperature resistivity of PVAc0.4/EVA0.6/GP-CNF at different CNF contents.

Figure 4 shows the room temperature resistivity of PVAc0.4/EVA0.6/GP-CNF with different levels of CNF addition (errors are given in Supporting Information). The room temperature resistivity increased by three orders of magnitude after adding only 1 wt.% CNF or 2 wt.% CNF compared to that without CNF. As the CNF content continued to increase, the room temperature resistivity decreased and then increased, reaching a minimum value of 205.4 Ω·m at 3 wt.% CNF, approximately twice the resistivity without the addition of CNF. The reason for this trend may be that when 1 wt.% CNF or 2 wt.% CNF is added, the amount of CNF is too small not only to form a new conductive chain containing more CNF, but also the insertion of CNF may affect the van der Waals forces between graphite [49] and thus destroy the graphite–graphite conductive network, resulting in a higher resistivity at this time. With the addition of more CNF, CNF starts to participate in the composition of the conducting network, and graphite–CNF–graphite and CNF–CNF conducting channels appear. However, continuing to increase the CNF content after more than 3 wt.% will make it difficult to disperse the CNFs with high aspect ratios by entangling them with each other [40], and the CNF entanglement phenomenon can be observed in Figure 2e. The large amount of CNF agglomeration will reduce the utilization of CNF and make less CNF form the conductive network, and it will affect the composition of the full conductive network, so the continued addition of CNF will instead increase the resistivity.

**Figure 5:**
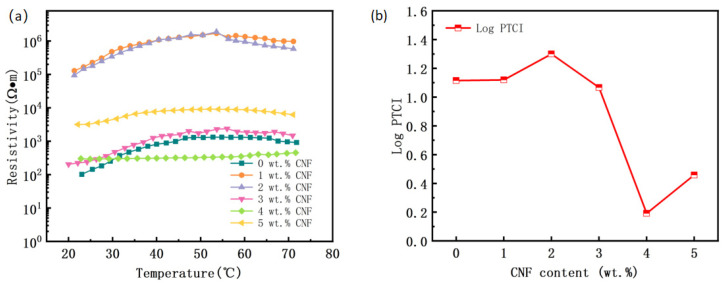
PVAc0.4/EVA0.6/GP-CNF composites with different CNF contents. (**a**) Resistance temperature characteristic curve; (**b**) PTC intensity.

The temperature resistance characteristic curves of the PVAc0.4/EVA0.6/GP-CNF composites are depicted in Figure 5. When the CNF content is low, the PTC strength is similar to that without CNF addition. The PTC curves at 1 wt.% and 2 wt.% CNF content are similar, suggesting that the conductive network within the material may be comparable at this point and inferior to the graphite conductive network without CNF, resulting in a higher room temperature resistivity. At 3 wt.% CNF content, the PTC strength and room temperature resistivity are closest to those without addition, and there is almost no NTC phenomenon, indicating potential good PTC repeatability. As the CNF content increases, the PTC strength decreases, with 4 wt.% and 5 wt.% CNF exhibiting weaker PTC effects. This is attributed to the rise in conductive particles within the matrix, enhancing the conductive particle percentage and conductive channel formation probability. During warming, conductive fillers may be displaced from their equilibrium positions, moving closer to other conductive particles, enabling the formation of new conductive channels. This second filler addition partially negates the PTC effect. The high CNF content facilitates easier reconfiguration of the conductive network during warming after deconstruction.

**Figure 6:**
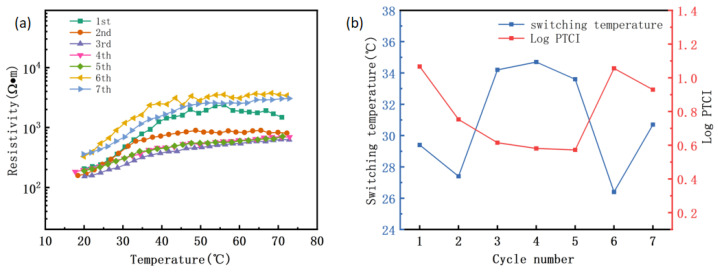
PVAc0.4/EVA0.6/GP-3wt.%CNF composite heating cycle. (**a**) Resistance temperature characteristic curve; (**b**) Curie temperature and PTC intensity.

Collectively, it seems that the composite with 3 wt.% CNF has the best PTC performance. Therefore, the PTC cycling curve of PVAc0.4/EVA0.6/GP-3wt.%CNF was further tested to investigate its PTC reproducibility. From Figure 6a, it was found that the material exhibits a very weak NTC effect only during the first heating, and the NTC effect disappears during subsequent heating. It can even be observed that the trend at the end of the curve is gradually upward, with only the first curve ending slightly downward. The second curve shows a long “plateau” after 50 °C, and the third to fifth curves are very similar and no longer have a “plateau”. However, the end of the curve goes up, indicating that the PTC effect of the material continues from room temperature to the test cutoff temperature. The sixth and seventh cycles also maintain this pattern, with the difference being that the room temperature resistivity remains largely unchanged for the first five cycles but slightly increases for the last two. The gradual elimination of the NTC effect and even the transition to a PTC effect with increasing heating times is an interesting phenomenon, as it is contrary to the results and patterns of many studies [27,50]. This indicates that the PTC of this material is reproducible and does not deteriorate quickly with increasing use. The significant increase in resistivity in the sixth and seventh cycles compared to previous cycles may be attributed to irrecoverable changes in the structure, volume, aggregation state, and distribution of the conductive fillers in the material after the sixth cycle. This can also be supported by the changes in Curie temperature [51]. From Figure 6b, it can be observed that the Curie temperature is similar for the third, fourth, and fifth cycles, but significantly decreases for the sixth and seventh cycles. Since the Curie temperature is related to the phase transformation process of the composite [29], this change indicates that the material undergoes a different phase transformation in the latter two cycles compared to before.

The Curie temperature during the initial heating of PVAc0.4/EVA0.6/GP-3wt.%CNF increased by approximately 1 °C compared to the material without CNF, reaching 29.4 °C, and remained at approximately 30 °C during cyclic heating. The PTC strength decreased with the number of cycles during the first few cycles, and increased to values close to the initial during the last two cycles. Taken together, it appears that this material has excellent PTC repeatability, which also indicates its potential for long-term use.

**Figure 7:**
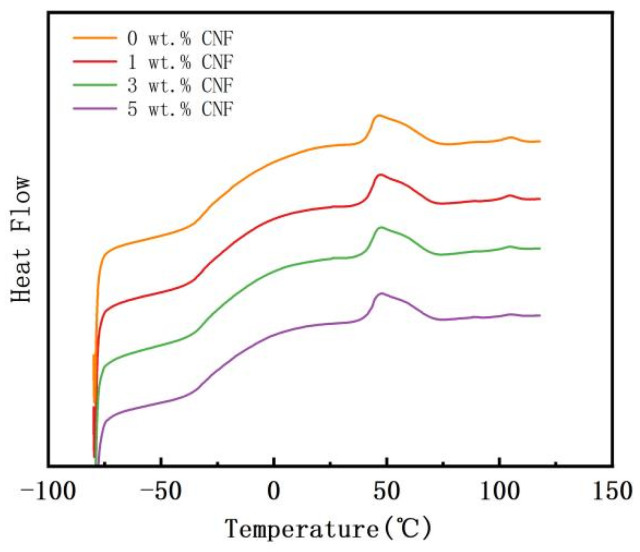
DSC curves of PVAc0.4/EVA0.6/GP-CNF at different CNF contents.

To investigate the effect of CNF addition on the melting point and glass transition temperature of the polymer matrix, PVAc0.4/EVA0.6/GP without CNF and three typical concentrations of the material with CNF added were selected for DSC testing. The results are shown in Figure 7. It can be seen that the addition of CNF has essentially no effect on the glass transition temperature and melting temperature of the copolymer matrix, which are around −28 °C for these samples, while the melting peaks at 51 °C and 105 °C also largely overlap. However, with an increase in CNF content, the peak height of the melt peak at 105 °C gradually decreased, indicating that a high content of CNF would affect the formation of crystal structures in EVA and reduce its crystallinity.

Additional details regarding this Section 3.2 can be found in the Supporting Information (Figures S2–S5 and the related discussions in Supporting Information).

### 3.3. Impact of Mixed Fillers on Self-Limiting Performance

Based on the results of previous PTC performance tests of the composites, the PVAc0.4/EVA0.6/GP-3wt.%CNF composite was selected as the main object for the next Joule heating performance tests.

(1) Room temperature ambient heating test

**Figure 8:**
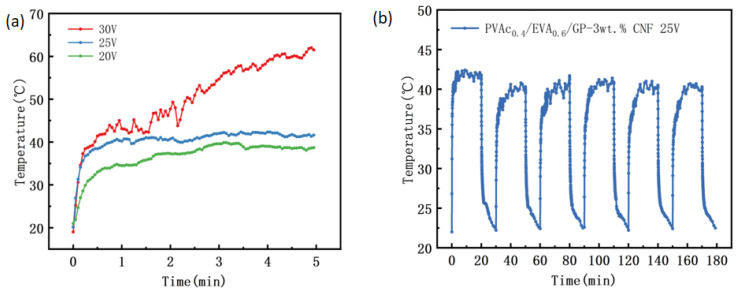
Self-limiting heating of PVAc0.4/EVA0.6/GP-3wt.% CNF composites. (**a**) Temperature-time curves at 20 V, 25 V, and 30 V; (**b**) cyclic heating-cooling test at 25 V cyclic voltage.

From Figure 8a, it can be seen that with an increase in voltage, the material gradually reaches the self-limiting temperature. At 20 V and 25 V, the material shows good self-limiting ability, reaching the self-limiting temperature in approximately 1 min. After this, the temperature increases slowly with time to reach equilibrium temperatures of 42 °C and 40 °C, respectively. However, when the voltage is 30 V, the temperature of the material rises rapidly to 40 °C and then increases to 61 °C due to the large heating power, indicating a lack of good self-limiting ability.

To evaluate its electrical heating cycle stability and compare it with the previous material, it was chosen to apply 25 V for six electrical heating tests, and the results are presented in Figure 8b. The initial heating power was 3.04 W, and the power density was 3.04 W/cm^2^, approximately six times higher than that of the PS0.4/EVA0.6/GP material and half of the PVAc0.4/EVA0.6/GP material. It is observed that the equilibrium temperature during the first heating was approximately 42 °C. The subsequent five heating processes involved a period of temperature fluctuations within a specific range, not exceeding 2 °C overall, and stabilizing near 40 °C. Although the equilibrium temperature during the last five heating cycles was lower than the first one, the lowest equilibrium temperature was only reduced by approximately 2 °C compared to the first, demonstrating excellent electrical heating stability. This indicates that, although the initial heating power decreases after adding CNF, the electrical heating stability of the composite significantly improves, enhancing its overall self-limiting heating performance. This has a significant impact on the practical application of the material and provides a reference solution to the problem of short service life in low-temperature polymer-based PTC materials, suggesting that this material has great potential for use in low-temperature heating and temperature control applications.

(2) Low-temperature environment heating test

In order to test the electrical heating performance of the material in a low-temperature environment where it can freeze, we cut a PVAc0.4/EVA0.6/GP-3wt.%CNF sample to a size of 20 mm × 20 mm, and coated both sides of the sample with silver paste. Then, the sample was covered with copper foil as the electrode, the DC voltage source used before was connected, and the sample was heated at different voltages, and its temperature variation with time was plotted. The graphs were plotted against time. In this experiment, the heating was started from an ambient temperature of about −10 °C.

**Figure 9:**
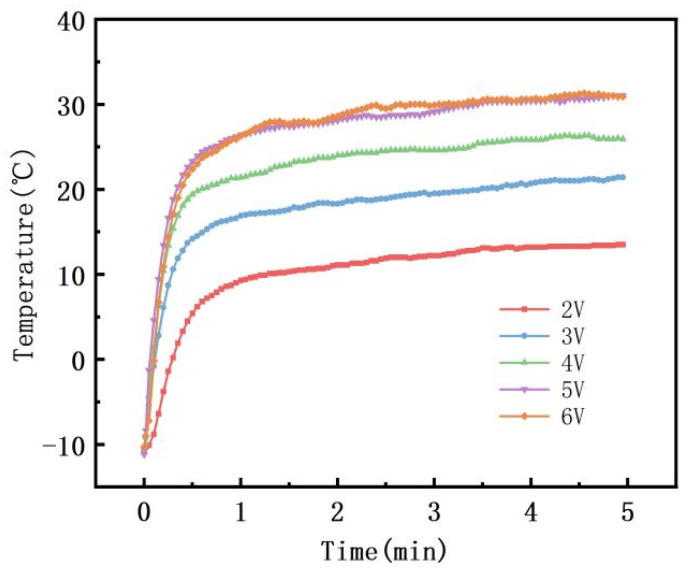
Electrical heating performance test of PVAc0.4/EVA0.6/GP-3wt.%CNF at different voltages at −10 °C.

As the applied voltage increased and the heating power density increases, the heating rate and the equilibrium temperature of the sample gradually increase (Figure 9). When the applied voltage is less than 5 V, the sample temperature cannot reach the Curie temperature due to the low heating power. When the applied voltage reaches 5 V and above, it takes only about 1 min to reach the equilibrium temperature and keep it around 30 °C, i.e., it remains stable near the Curie temperature. This equilibrium temperature is lower than the equilibrium temperature achieved at room temperature, as the low-temperature environment accelerates the heat dissipation rate of the material, ultimately resulting in a lower equilibrium temperature. The 5 V and 6 V heating curves are closer and show similar heating patterns. In the initial stage, due to the low resistivity, the heating power is high, resulting in a fast heating rate and rapid temperature rise. However, as the Curie temperature is approached, the resistivity of the material rises significantly, leading to a decrease in heating power and a subsequent decrease in the heating rate, thus maintaining the equilibrium temperature near the Curie temperature. In the subsequent heating process, the resistance fluctuates with temperature, but the heating power is automatically adjusted. The material will automatically reduce the power when overheating and increase the power when cooling, ultimately achieving the self-limiting capability of the material. Given this, at these voltages, PVAc0.4/EVA0.6/GP-3wt.%CNF exhibits good automatic temperature control in low-temperature environments, indicating its potential application in the field of de-icing.

(3) Breakage heating test

**Figure 10:**
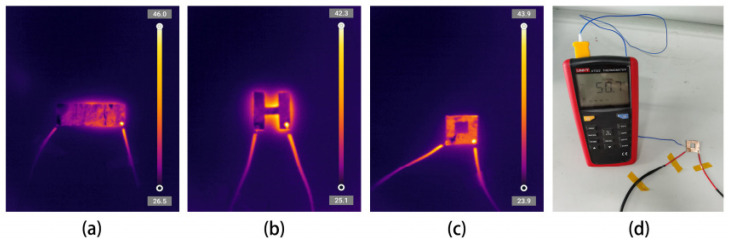
Infrared imaging of PVAc0.4/EVA0.6/GP-3wt.%CNF composite heated in different states: (**a**) rectangle; (**b**) “H” shape; (**c**) “back” shape; (**d**) thermocouple test of the surface temperature of the “H” shaped sample.

The heating performance of PVAc0.4/EVA0.6/GP-3wt.%CNF on complex surfaces is shown in Figure 10, with the same upper electrode as in the low temperature environment test. The infrared images of the material in the rectangular, “H”, and “back” heating states are shown in the figure.

It is found that the material can be heated successfully in intact, complex, and broken states, and the equilibrium temperature is over 40 °C. Since the surface of the material is covered with a silver paste layer and a copper foil layer, and the metal blocks infrared radiation, the infrared image cannot fully show the internal heat distribution of the material. However, based on the infrared image, the surface and edge of the material are warmer than the ambient temperature (25 °C), indicating that the damaged sample can still be heated normally. Figure 10d shows the surface temperature of the sample directly tested with a thermocouple, reaching 50 °C, which also confirms the heating function of the damaged sample. This is due to the use of a double-sided electrode, and the partial damage of the sample does not affect the heating performance in other areas, demonstrating the reliability of the material in some actual harsh environments. If the heating material is broken due to external factors, this is a fatal issue for conventional metallic heating materials used in aircraft de-icing. The results also demonstrate that this material can be used in applications requiring irregular or complex shapes, where many heating materials have previously been unable to achieve heating on complex surfaces [52,53]. 

Additional details regarding this Section 3.3 can be found in the Supporting Information (Figure S6 and the related discussions in Supporting Information).

### 3.4. Effect of Mixed Filler on Mechanical Properties

**Figure 11:**
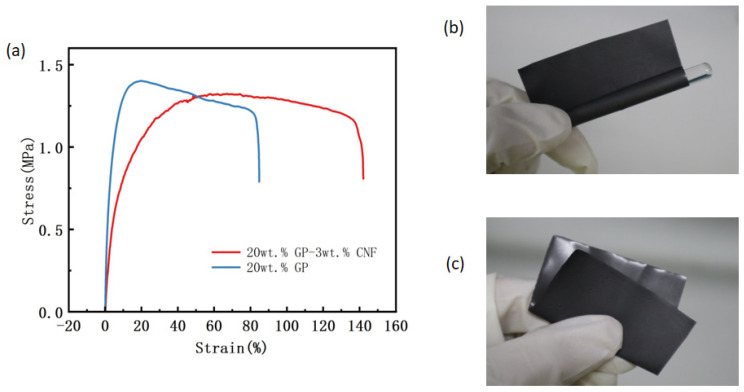
(**a**) Stress–strain curves of PVAc0.4/EVA0.6/GP and PVAc0.4/EVA0.6/GP-3wt.%CNF. PVAc0.4/EVA0.6/GP-3wt.%CNF (**b**) winding around glass rod and (**c**) folded physical diagram.

Figure 11a depicts the stress–strain curves of PVAc0.4/EVA0.6/GP and PVAc0.4/EVA0.6/GP-3wt.%CNF. Upon observing the two curves, it is evident that the strain at break of the material increased considerably from 85% to 142% after the addition of 3 wt.% CNF. This indicates a significant enhancement in the ductility of the material following the addition of the second-phase filler, CNF. However, the ultimate tensile strength of the material decreased slightly after the inclusion of CNF, suggesting a reduction in the stress at the onset of necking. The Young’s modulus of PVAc0.4/EVA0.6/GP-3wt.%CNF was calculated to be 9.24 MPa, and the toughness value was 1.68 MJ/m^3^ (while the Young’s modulus of PVAc0.4/EVA0.6/GP was calculated to be 7.92 MPa and the toughness value was 1.23 MJ/m^3^). This implies that the stresses required to induce elastic deformation in this material are relatively lower, and the composites with mixed fillers exhibit greater elasticity. Furthermore, the toughness value with the addition of CNF is higher than without it, demonstrating that the hybrid filler also enhances the toughness of the material. In summary, the blending of graphite and CNF fillers appears to have improved the overall mechanical properties of the composite.

Figure 11b,c show the physical images of PVAc0.4/EVA0.6/GP-3wt.%CNF wound and folded around a glass rod with a diameter of 5 mm, respectively, as can be seen from the figures. This film has very good flexibility and can be bent and folded at will, which can be applied to complex shapes or curved surfaces.

## 4. Conclusions

In this paper, PVAc0.4/EVA0.6/GP composites with varying CNF contents were prepared by incorporating CNF as a secondary conductive filler into the PVAc0.4/EVA0.6/GP composite with the best overall performance. This approach enhanced the stability of the conductive network and the thermal stability of the materials. The study also examined the impact of CNF content on the microscopic morphology, electrical conductivity, and PTC properties of the composites. Additionally, the Joule heating properties and mechanical properties of the optimal composition, PVAc0.4/EVA0.6/GP-3wt.%CNF, were tested at room and low temperatures. The PTC material exhibited a low switching temperature point (<30 °C), low room temperature resistivity (205.4 Ω·m), high PTC repeatability and flexibility, high heating stability and reliability, short heating response time, and excellent adaptive thermal control performance.

## Figures and Tables

**Table 1:** Surface free energies of the fillers and polymers.

Materials	Total Surface Energy γ (mJ·m^−2^)	Dispersion Component $\gamma^{d}$ (mJ·m^−2^)	Polar Component $\gamma^{p}$ (mJ·m^−2^)
EVA (40 wt.%VA)	35.9	32.5	3.4
PVAc	36.5	25.1	11.4
Graphite	52.8	41.1	11.7
CNF	94.7	92.1	2.6

**Table 2:** Interfacial energies as calculated using harmonic mean equation and geometric mean equation.

Materials	Interfacial Energy by Harmonic Mean Equation (mJ·m^−2^)	Interfacial Energy by Geometric Mean Equation (mJ·m^−2^)
EVA/Graphite	5.5	3.0
PVAc/Graphite	3.9	2.0
EVA/CNF	28.6	15.2
PVAc/CNF	43.8	24.1
EVA/PVAc	5.3	2.8

**Table 3:** Wetting coefficient and predicted location of graphite.

Blends	A	B	$\omega_{a}$ (Harmonic Mean Equation)	$\omega_{a}$ (Geometric Mean Equation)	Predicted Location
Graphite/PVAc/EVA	EVA	PVAc	0.3	0.4	PVAc/EVA interface
CNF/PVAc/EVA	EVA	PVAc	−2.9	−3.2	EVA

## Data Availability

The original contributions presented in the study are included in the article/Supplementary Material, further inquiries can be directed to the corresponding author/s.

## Supplementary Materials

The following supporting information can be downloaded at: https://www.mdpi.com/article/10.3390/polym16142028/s1, Figure S1. Cross section SEM images of PVAc_0.4_/EVA_0.6_/GP-CNF composites; Figure S2. Cross section SEM images of four different PVAc/EVA/GP composite; Figure S3. EDS diagrams of the two composite materials; Figure S4. Cross section SEM images of PVAc_0.4_/EVA_0.6_/GP-CNF composites after heating; Figure S5. FTIR result of PVAc_0.4_/EVA_0.6_/GP-CNF composites; Figure S6. (a) Influence of humidity on resistance of the composites, (b) application of the composites as temperature sensor on skin.
