# Supplementary material for: Flexible Positive Temperature Coefficient Composites (PVAc/EVA/GP-CNF) with Room Temperature Curie Point
Source: Polymers (Basel). 2024 Jul 16;16(14):2028. doi: 10.3390/polym16142028 (PMC11280720; doi:10.3390/polym16142028)
Supplement: Supplementary file 1 [file polymers-16-02028-s001.zip › polymers-3051994-supplementary.pdf]

Supporting Information

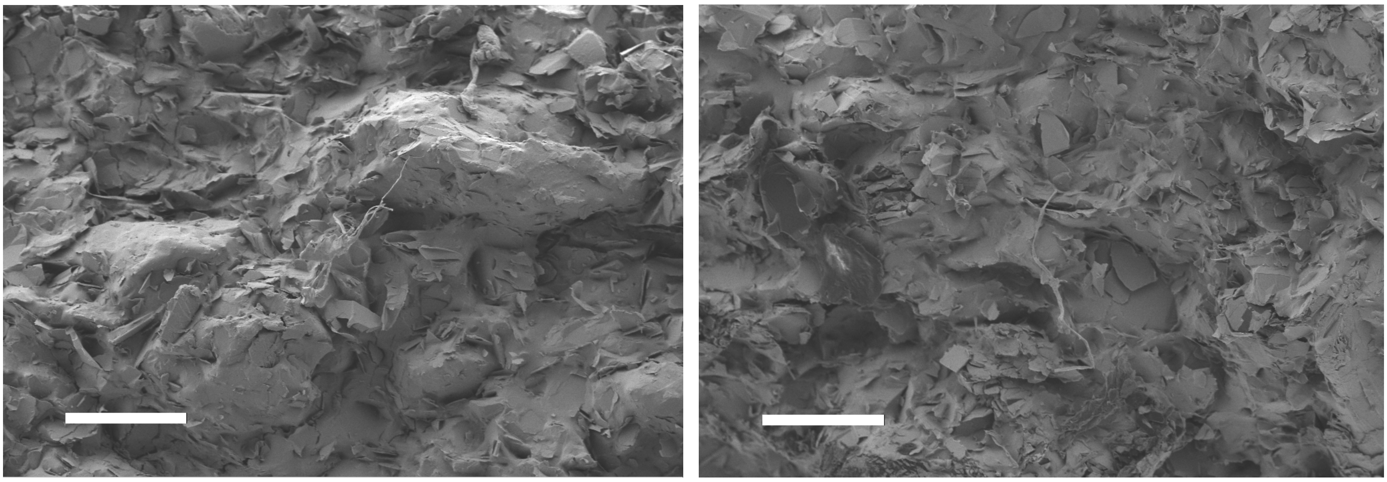

Figure S1. Cross section SEM images of $PVAc_{0.4}/EVA_{0.6}$/GP-CNF composites. Scale bar: 20 μm.

Errors of Figure 4:

1) Samples with 0 wt.% CNF: 95.2-98.9 Ω·m.
2) Samples with 1 wt.% CNF: 100.5- 103.2 kΩ·m.
3) Samples with 2 wt.% CNF: 88.7-91.3 kΩ·m.
4) Samples with 3 wt.% CNF: 201.3-209.6 Ω·m.
5) Samples with 4 wt.% CNF: 293.2-307.4 Ω·m.
6) Samples with 5 wt.% CNF: 1.95-2.06 kΩ·m.

Figures S2 (a-d) show SEM images of cross-sections of PVAc/EVA/GP composite materials with different mass ratios. Given that the VA group content in EVA is 40 wt.%, and based on the mass ratio of PVAc to EVA in $PVAc_{0.4}/EVA_{0.6}$/GP composite materials, it can be calculated that the oxygen element in $PVAc_{0.4}/EVA_{0.6}$/GP composite materials accounts for 62.5% of the total oxygen element content. In the $PVAc_{0.75}/EVA_{0.25}$/GP composite material, the oxygen element in PVAc accounts for 88.3% of the total amount.

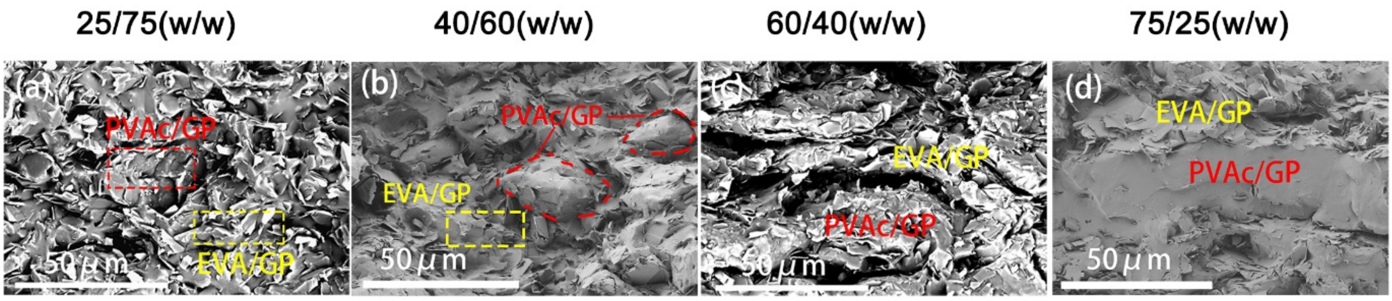

Figure S2. Cross section SEM images of four different PVAc/EVA/GP composite: (a) $PVAc_{0.25}/EVA_{0.75}/GP$, (b) $PVAc_{0.4}/EVA_{0.6}/GP$, (c) $PVAc_{0.6}/EVA_{0.4}/GP$, (d) $PVAc_{0.75}/EVA_{0.25}/GP$.

Based on the EDS diagrams of the two composite materials (Figure S3), it can be inferred that the green part in Figures S3 (a) and (b) is mainly PVAc. Two types of polymers have been marked with different colors in Figure S2. When the proportion of PVAc to the matrix polymer is less than or equal to 60 wt.%, the PVAc/EVA/GP composite material exhibits a co-continuous biphasic polymer (Figure S2 (a-c)); When PVAc accounts for 75 wt.% of the matrix, the binary phase undergoes layering, with PVAc in the lower layer (Figure S2 (d)). From Figure S2 (a-d), it can be observed that graphite is uniformly distributed throughout the matrix, and a large amount of graphite can be seen in both EVA and PVAc phases.

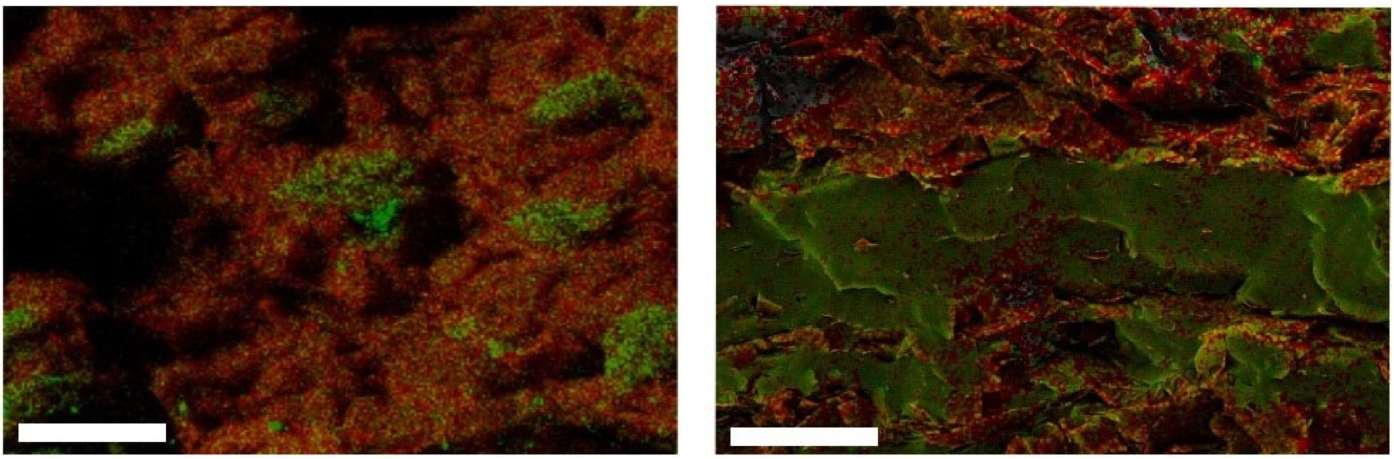

Figure S3. EDS diagrams of the two composite materials (left: $PVAc_{0.4}/EVA_{0.6}/GP$, right: $PVAc_{0.75}/EVA_{0.25}/GP$).

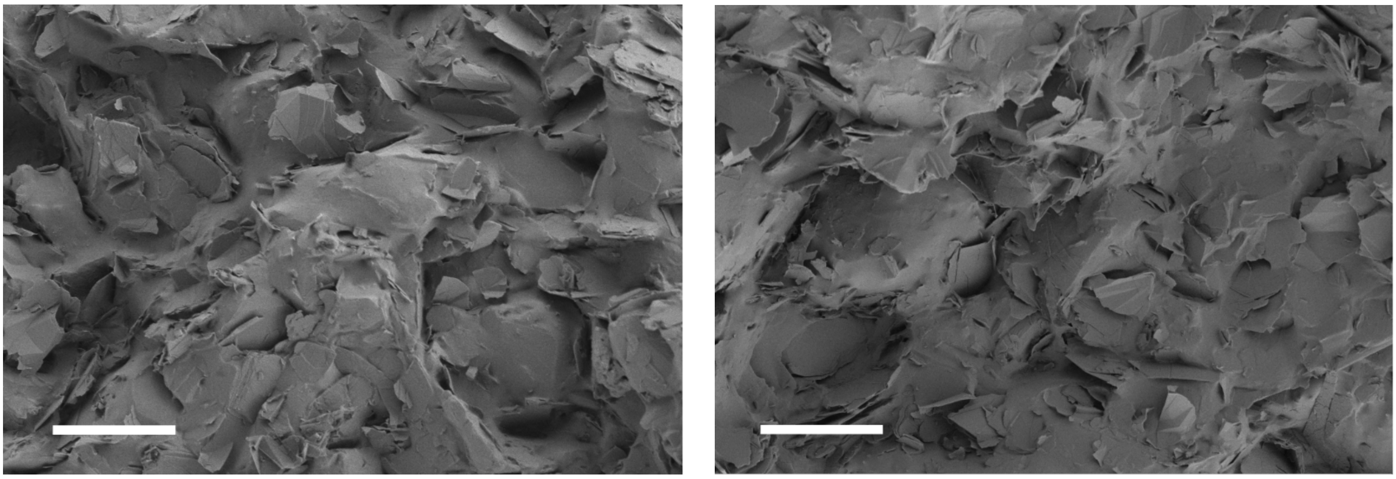

Figure S4. Cross section SEM images of PVAc0.4/EVA0.6/GP-CNF composites after 6 heating cycles (left) and 7 heating cycles (right). Scale bar: 20 μm.

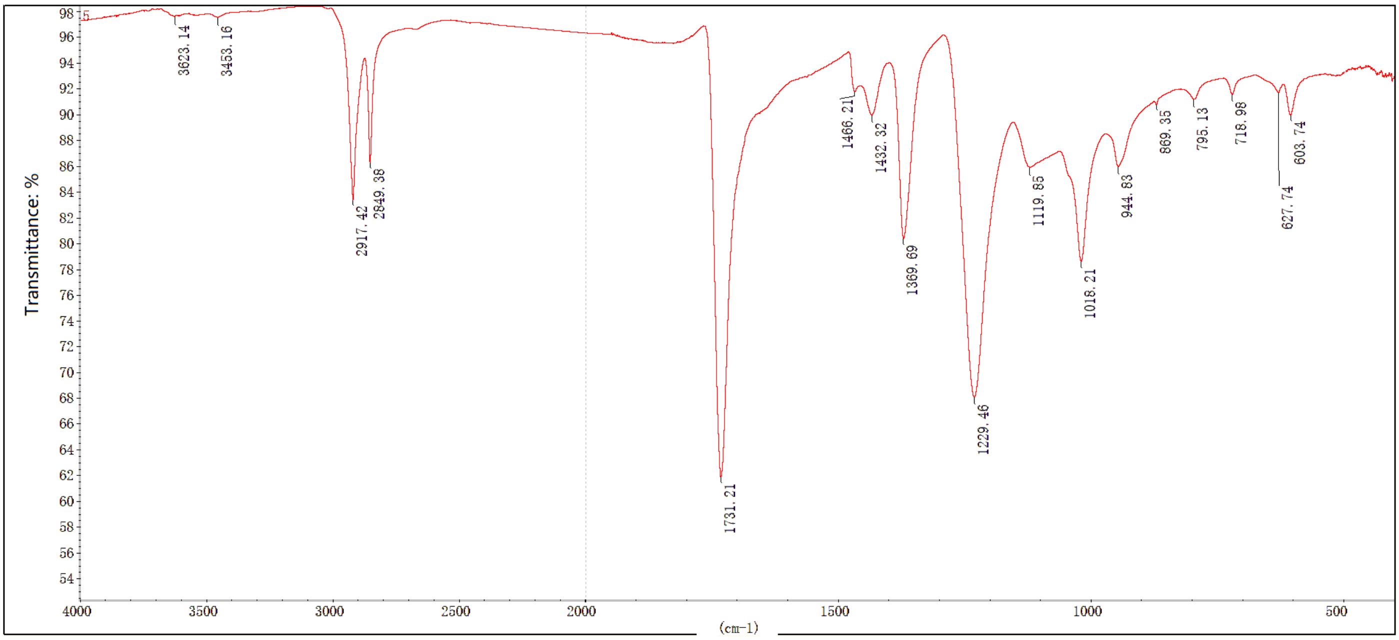

Figure S5. FTIR result of PVAc0.4/EVA0.6/GP-CNF composites.

Humidity is very important to the electrical stability of the composites. We detect the influence of humidity in the environment on the composites, the humidity environment is created by configuring a saturated saline solution in a jar. In this experiment, our humidity environment is 84.2% RH, which is made up of KCl and deionized water. In order to highlight the role of encapsulation, composite films without encapsulation and double-layer encapsulated are suspended in a jar with a humidity environment. The films

were in a closed space, and only two wires are drawn for measurement. Since the films were pasted on the surface of the object when it is applied, the back is isolated from the air. As shown in Figure S6(a), it can be seen the resistance of film without encapsulation in a humidity environment increases significantly with time, but encapsulated film can resist the influence of humidity, and its resistance remained almost unchanged, which indicating that the encapsulation has good airtightness. After encapsulated, we can use our sample to make temperature sensors which can detect human temperature, as shown in Figure S6(b).

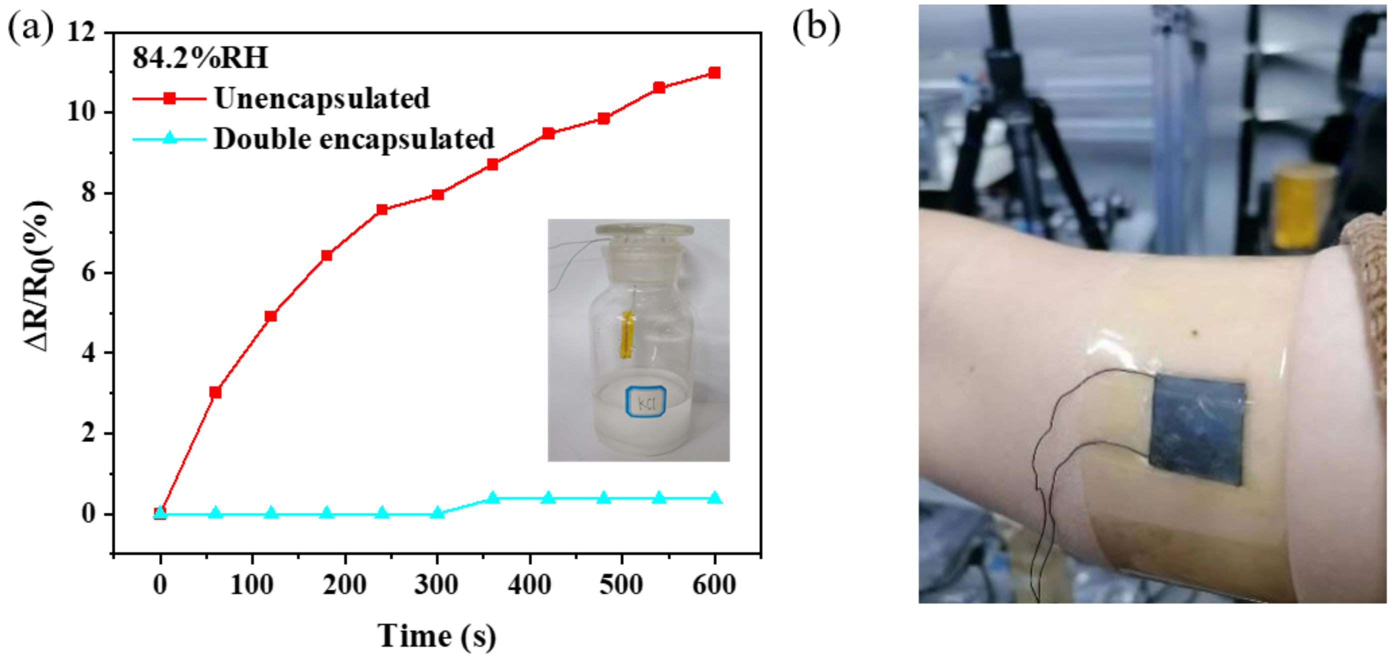

Figure S6. (a) Influence of humidity on resistance of the composites, (b) application of the composites as temperature sensor on skin.
